# Oral Semaglutide in Routine Clinical Practice: 24‐Month Real‐World Effectiveness, Persistence and Safety in Adults With Type 2 Diabetes

**DOI:** 10.1111/dom.71371

**Published:** 2026-09-21

**Authors:** Maria Pompea Antonia Baldassarre, Giulia Di Dalmazi, Federica Carrieri, Giorgia Centorame, Luigi Piacentino, Sara Coluzzi, Gloria Formoso

**Affiliations:** ^1^ Department of Medicine and Aging Sciences G. D'Annunzio University of Chieti‐Pescara Chieti Italy; ^2^ Center for Advanced Studies and Technology (CAST) G. D'Annunzio University of Chieti‐Pescara Chieti Italy; ^3^ Endocrinology and Metabolic Disease Clinic of Pescara Pescara Italy

**Keywords:** GLP‐1 receptor agonist, oral semaglutide, real‐world evidence, treatment persistence, type 2 diabetes

## Abstract

**Aims:**

Real‐world long‐term data on oral semaglutide are limited. We evaluated its 24‐month effectiveness, persistence, and safety in adults with type 2 diabetes (T2D).

**Materials and Methods:**

This retrospective real‐world study included 232 adults with T2D initiating oral semaglutide at an Italian diabetes clinic. Changes in HbA1c, body weight, eGFR, urinary albumin‐to‐creatinine ratio (UACR) and lipid profile were assessed using linear mixed models. The composite endpoint of HbA1c ≤ 7% and ≥ 5% weight loss was assessed over follow‐up. Treatment persistence was evaluated by Kaplan–Meier analysis, and predictors of discontinuation by multivariable Cox regression with time‐dependent covariates. HbA1c and body weight before and after switching to another GLP1 receptor agonist were also evaluated.

**Results:**

Over 24 months, oral semaglutide significantly reduced HbA1c (−0.8%; *p* < 0.0001) and body weight (−5.7 kg; *p* < 0.0001). The composite endpoint was progressively achieved by 44.0% of individuals at 24 months. Non‐breakfast administration was independently associated with lower HbA1c after adjustment for BMI, age and sex (*β* = −0.32; *p* = 0.041). eGFR and UACR remained stable while total and LDL cholesterol decreased significantly (both *p* < 0.0001). Treatment persistence at 24 months was 68.5% (mean 19.4 months). Among discontinuers, switching to another GLP1 receptor agonist was the most common strategy (36.2%) and resulted in a further HbA1c reduction (Δ = −0.98%; *p* = 0.005). No safety concerns emerged.

**Conclusions:**

In this real‐world cohort, oral semaglutide provided sustained improvements in glycaemic control, body weight, and lipid profile, with preserved renal function and high treatment persistence over 24 months.

## 1 Introduction

Semaglutide is the only once‐daily oral glucagon‐like peptide‐1 receptor agonist (GLP‐1RA) currently available for the treatment of type 2 diabetes (T2D). Randomised controlled trials have demonstrated its efficacy in improving glycaemic control and reducing body weight, with a safety profile consistent with the GLP‐1RA class [1]. The SOUL cardiovascular outcomes trial confirmed the cardiovascular benefit of oral semaglutide, showing a 14% relative risk reduction in major adverse cardiovascular events compared with placebo (hazard ratio 0.86; 95% CI 0.77–0.96; *p* = 0.006) over a median follow‐up of 49.5 months [2]. Rates of serious adverse events and gastrointestinal disorders were comparable between treatment groups, supporting the overall safety of oral semaglutide [2].

Real‐world data on the long‐term effectiveness and tolerability of oral semaglutide are still scarce. In our previous work [3], we evaluated its use after 6 months of treatment in routine clinical practice and found significant improvements in HbA1c and body weight, although 93% of individuals were treated with doses below the maximum of 14 mg daily.

The present study extends these observations by evaluating the 2‐year effectiveness, safety, and treatment persistence of oral semaglutide in a real‐world cohort of adults with T2D.

## 2 Methods

This was a retrospective, observational real‐world study conducted at the Endocrinology and Metabolic Disease Clinic in Pescara, Italy. Data were obtained from the routinely used electronic medical record system (Metaclinic software, METEDA, San Benedetto del Tronto, Italy), which supports the standardised collection of clinical information in Italian diabetes outpatient clinics. The study was conducted in accordance with the principles of the Declaration of Helsinki and approved by the local Ethics Committee (Internal Review Board, University of L'Aquila; protocol number 48741, May 8th, 2023).

This study uses the method of Baldassarre et al. and the methods description partly reproduces their wording [3].

Adults with T2D who initiated oral semaglutide between January 2021 and December 2022 and had documented HbA1c and body weight values at baseline were included. Individuals with type 1, secondary, or gestational diabetes, or with missing baseline or follow‐up data, were excluded. No additional exclusion criteria were applied.

The following clinical and laboratory parameters were collected: age, sex, diabetes duration, height, body weight (BW), waist circumference (WC), systolic blood pressure, diastolic blood pressure, HbA1c, fasting plasma glucose (FPG), triglycerides, total cholesterol, high‐density lipoprotein cholesterol (HDL‐C), low‐density lipoprotein cholesterol (LDL‐C), serum creatinine, aspartate aminotransferase (AST), alanine aminotransferase (ALT), gamma‐glutamyl transferase (GGT), estimated glomerular filtration rate (eGFR), and urinary albumin‐to‐creatinine ratio (UACR). Information on diabetes complications, comorbidities, and concurrent medications was also retrieved. Microangiopathy was defined as the presence of at least one of the following: neuropathy, retinopathy, macular oedema, chronic kidney disease, or microalbuminuria. Macroangiopathy included any history of stroke or transient ischaemic attack, myocardial infarction, coronary or peripheral artery disease, or previous coronary/peripheral revascularisation procedures.

Oral semaglutide was prescribed during routine outpatient visits and initiated according to the approved dosing schedule, starting with 3 mg daily for 4 weeks, followed by an increase to 7 mg and up to 14 mg daily when necessary for glycaemic control optimisation and/or weight loss, if well tolerated. Individuals received standardised counselling on correct drug administration, including ingestion with minimal water, on an empty stomach, at least 30 min before meals or other medications. Follow‐up data were collected at approximately 6, 12, and 24 months after treatment initiation. At each visit, updated values of HbA1c, BW, and any reported adverse events were recorded. Reasons for treatment discontinuation were systematically documented and categorised (e.g., gastrointestinal intolerance, lack of efficacy, patient preference, or other clinical reasons).

Variables were tested for normality using the Shapiro–Wilk test. Continuous variables, with a normal distribution, are presented as mean values and standard deviation (SD), while variables not normally distributed are presented as median values and interquartile ranges (IQR). Categorical variables are expressed as frequencies and percentages (%).

Longitudinal linear mixed models for repeated measures were applied to assess trends over time in continuous outcomes (HbA1c, body weight, eGFR, total cholesterol, HDL cholesterol, triglycerides, LDL cholesterol). Longitudinal changes in HbA1c and body weight were assessed in men and women using time x sex interaction term to test for sex‐specific trajectories.

The effect of administration timing (before breakfast vs. before lunch/dinner) on longitudinal HbA1c was assessed using a linear mixed‐effects model adjusted for age, sex and baseline BMI. Results were expressed as the estimated mean change from baseline with their 95% confidence intervals (95% CI). The composite endpoint of HbA1c ≤ 7% and weight loss ≥ 5% was evaluated at 6, 12 and 24 months on individuals still receiving oral semaglutide at each timepoint and with both HbA1c and body weight measurements available; discontinuations and switches were excluded from analysis.

Kaplan–Meier survival analyses were used to assess treatment persistence at 24 months in individuals receiving oral semaglutide. Only treatment discontinuations occurring within the first 24 months after oral semaglutide initiation were considered as events. A multivariable Cox proportional hazards model incorporating time‐dependent covariates was applied to account for non‐proportional hazards and to evaluate potential factors affecting treatment persistence. In individuals switching to another glucose‐lowering therapy after discontinuation, pre‐ and post‐switch HbA1c and body weight were compared using paired *t*‐tests, and longitudinal trajectories in switchers versus non‐switchers were assessed with mixed‐effects models.

All statistical analyses were performed using Stata statistical software, released 17.0 (Stata Corp., College Station, TX). A *p*‐value < 0.05 was considered statistically significant.

## 3 Results

### 3.1 Population Characteristics

Baseline characteristics are summarised in Table 1.

**TABLE 1:** Characteristics of study individuals.

Variables	Value
Sex, male	130 (56)
Age, years	67 (15)
Diabetes Duration, years	9 (12)
Body weight, kg	82 (25)
BMI, kg/m^2^	30.1 (7.1)
Systolic blood pressure, mmHg	139 (20)
Diastolic blood pressure, mmHg	78.5 (18)
Laboratory data	
HbA1c, %	7.6 (1.1)
HbA1c, mmol/mol	59 (12)
Total cholesterol, mg/dL	175 (42.6)
HDL cholesterol, mg/dL	46 (17)
LDL cholesterol, mg/dL	98.9 (38.8)
Triglycerides, mg/dL	158 (97.5)
eGFR mL/min/1.73 m^2^	80.5 (31.7)
UACR, mg/g	8.8 (26.4)
Comorbidities	
Hypertension	152 (65.5)
Dyslipidemia	151 (65.1)
Coronary heart disease	32 (13.8)
CKD stage III	37/173 (21.4)
Retinopathy	33 (14.2)
Any microangiopathy	85 (36.6)
Any macroangiopathy	68 (29.3)
Diabetes background medications	
Only diet	14 (6)
Metformin	200 (91.7)
SGLT‐2 inhibitors	89 (40.8)
DPP‐4 inhibitors	52 (23.9)
Basal insulin	24 (11)
Pioglitazone	19 (8.7)
GLP1‐RA	19 (8.7)
Sulphonylureas	11 (4.7)
Other therapies	
Statin	122 (52.6)
RAS blockers	120 (51.7)
Beta‐blockers	85 (36.7)
Diuretics	59 (25.4)
Calcium channel blockers	52 (22.4)
Anti‐platelet agents	83 (35.8)
Anticoagulants	13 (5.6)

*Note:* Data are presented as median and interquartile range (IQR) or as number and percentage (%).

Abbreviations: CKD, chronic kidney disease (defined as eGFR < 60 mL/min/1.73 m^2^); eGFR, estimated glomerular filtration rate; RAS, renin angiotensin system; SGLT‐2, sodium glucose co‐transporter‐2; UACR, urinary albumin‐to‐creatinine ratio.

A total of 232 individuals (56% males, median age 67 years) were included in the study. At baseline, subjects presented an inadequate glycaemic control [HbA1c 7.6% (1.1)], obesity [BMI was 30.1 kg/m^2^ (7.1)] and long diabetes duration [9 years (12)]; data expressed as median (IQR). In 78.6% of subjects eGFR was greater than 60 mL/min/1.73 m^2^, while in less than 1% eGFR was lower than 30 mL/min/1.73 m^2^.

Most frequent comorbidities were hypertension (65.5%), dyslipidaemia (65.1%), microangiopathy (36.6%) or macroangiopathy (29.3%).

At the time of initiation of oral semaglutide, 91.7% of individuals were on metformin, 40.8% on SGLT‐2 inhibitors, 23.9% on DPP‐4 inhibitors, 8.7% on pioglitazone, 5.1% on a sulphonylurea, and 11% on basal insulin. Most patients were naïve to GLP1‐RA (91.3%), and only 8.7% of patients were switching from an injectable GLP‐1RA (3.2% from semaglutide once‐weekly, 2.3% from dulaglutide, 2.3% from exenatide, and 0.9% from liraglutide).

### 3.2 Oral Semaglutide Dose and Administration

Oral semaglutide was initiated at the recommended starting dose of 3 mg/day in 99.1% of individuals. At first available follow‐up (4.5 ± 1.6 months), the dose was titrated up to 7 mg/day in 85% of subjects, 10% remained at the 3 mg/day dose, and 3% escalated to 14 mg/day. At the final follow‐up (27.7 ± 3.8 months), half of the population still on therapy was receiving the highest dose of 14 mg (53.6%). These individuals showed a higher weight at baseline (88.6 ± 19.8 kg vs. 82.3 ± 18.6 kg in the non–14 mg group, *p* < 0.05).

### 3.3 Changes in HbA1c and Body Weight

After 24 months, the estimated mean changes from baseline in HbA1c and body weight were −0.8% (95% CI −1.00 to −0.59; *p* < 0.0001) and −5.7 kg (95% CI −7.61 to −3.75; *p* < 0.0001), respectively (Figure 1), with similar longitudinal patterns in men and women (data not shown).

**FIGURE 1:**
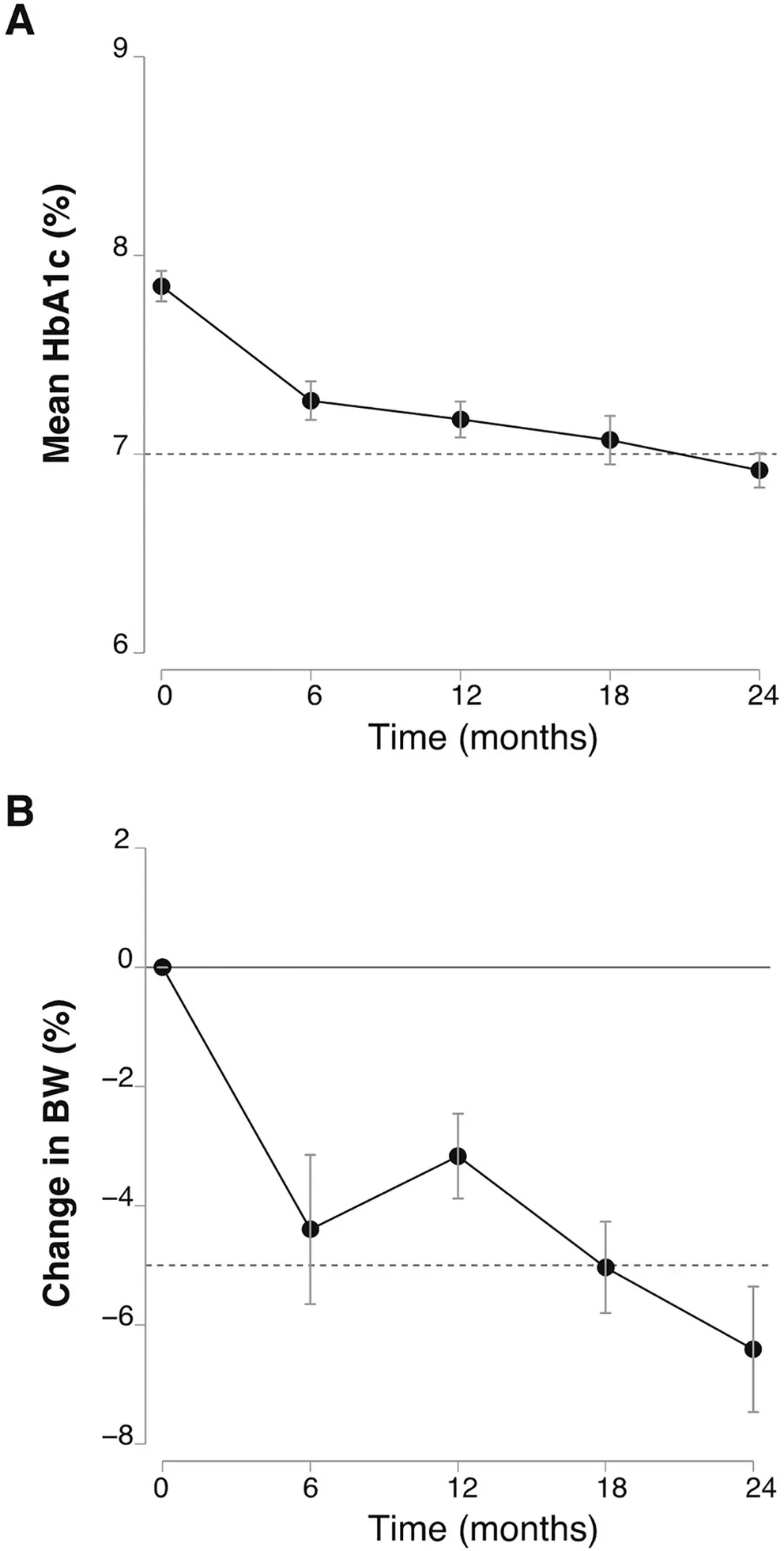
Effectiveness on HbA1c and body weight (BW). (A) Mean HbA1c (%) from baseline to 24 months. (B) The percent change in body weight from baseline to 24 months.

When individuals were stratified according to the maximum semaglutide dose achieved (14 mg/day vs. non‐14 mg/day), both groups showed significant reductions in HbA1c and body weight, with no significant time‐by‐treatment interaction.

### 3.4 Composite Endpoint of Glycaemic Control and Weight Reduction

Among individuals still receiving oral semaglutide at each visit, the proportion of individuals achieving the composite endpoint of HbA1c ≤ 7% combined with a body weight loss ≥ 5% from baseline progressively increased over the follow‐up period, from 23.9% (48/201) at 6 months to 24.5% (45/184) at 12 months and 44.0% (70/159) at 24 months.

The two individual components followed a similar pattern: the proportion of individuals achieving HbA1c ≤ 7% increased from 46.7% at 6 months to 47.9% at 12 months and 68.2% at 24 months, while the proportion of those achieving a body weight loss ≥ 5% remained stable from 6 to 12 months (33.3%) and increased up to 53.3% at 24 months.

### 3.5 Effect of Dose Timing on Glycaemic Response

Most individuals took oral semaglutide before breakfast (82.8%), while a minority took the drug before lunch (less than 1% of cases) or before dinner (in 16.4% of cases). In the linear mixed‐effects model with a random intercept for individual, non‐breakfast administration was associated with significantly lower HbA1c values compared with breakfast administration over the 24‐month follow‐up (*β* = −0.37; 95% CI −0.66 to −0.09; *p* = 0.010). This effect remained significant after adjustment for baseline BMI, age, and sex (*β* = −0.32; 95% CI −0.62 to −0.01; *p* = 0.041).

### 3.6 Changes in Renal Function and Cardiometabolic Parameters

eGFR remained essentially unchanged over the study period, with an estimated mean change from baseline at 24 months of −0.28 mL/min/1.73 m^2^ (95% CI −0.72 to +0.16; *p* = 0.222). UACR showed no significant variation over the 24‐month follow‐up; in the linear mixed model with random intercept for individual, the estimated mean change from baseline at 24 months was −11.94 mg/g (95% CI −35.06 to +11.17; *p* = 0.311).

Mean total and LDL cholesterol significantly decreased from baseline to the last follow‐up, with mean changes of −18.3 mg/dL (95% CI −25.83 to −10.81; *p* < 0.0001) and −17.1 mg/dL (95% CI −24.77 to −9.43; *p* < 0.0001), respectively. No statistically significant change in HDL cholesterol or triglycerides was reported over time. At 24 months, mean systolic and diastolic blood pressure showed small, non‐significant reductions from 139 (20) to 136 (19) mmHg and from 78.5 (18) to 77.4 (17) mmHg, respectively.

### 3.7 Background Glucose‐Lowering Therapy

Changes in background glucose‐lowering therapy over the follow‐up period are summarised in Figure 2. The proportion of individuals receiving SGLT‐2 inhibitors increased over the course of follow‐up (from approximately 40% at baseline to 50% at 24 months), while the use of other concomitant glucose‐lowering agents remained largely unchanged. The exception was DPP‐4 inhibitors, which were discontinued in all individuals at the time of oral semaglutide initiation, consistent with the known pharmacological overlap between the two drug classes.

**FIGURE 2:**
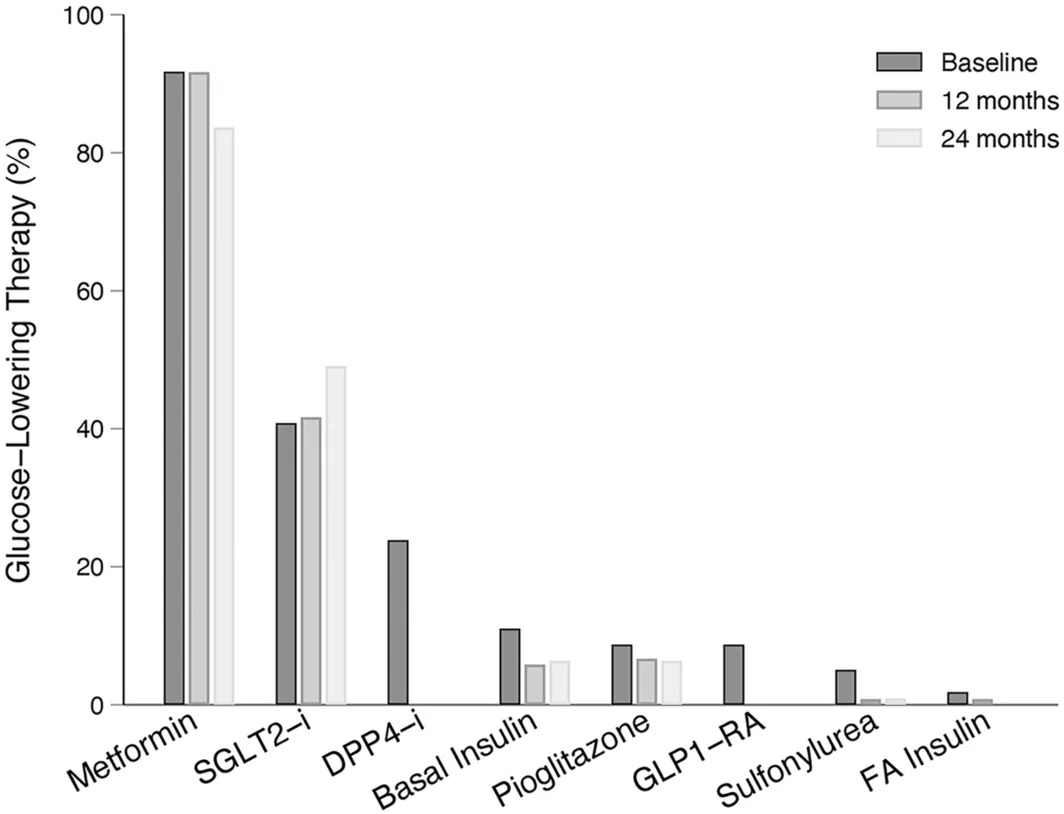
Changes in background glucose‐lowering therapy over the follow‐up period. DPP4‐i, dipeptidyl peptidase‐4 inhibitors; FA insulin, fast‐acting insulin; GLP1‐RA, glucagon‐like peptide‐1 receptor agonists; SGLT2‐i, sodium/glucose cotransporter‐2 inhibitors.

### 3.8 Treatment Persistence

At 24 months, 68.5% of individuals were still on oral semaglutide and the mean estimated persistence time was 19.4 months as shown in Figure 3.

**FIGURE 3:**
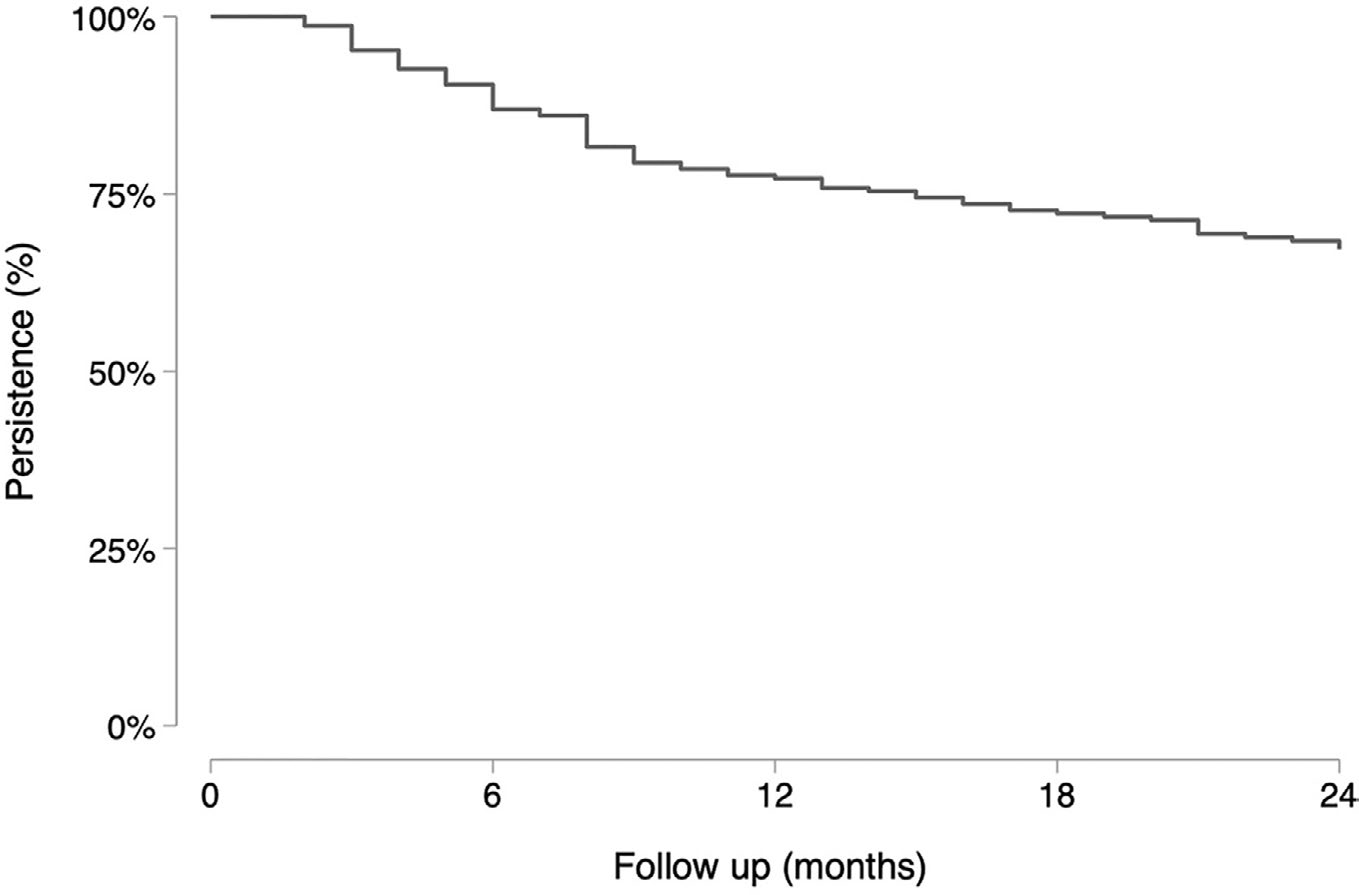
Kaplan–Meier curve for treatment persistence showing the proportion of individuals remaining on oral semaglutide over time.

To investigate factors associated with treatment persistence, a multivariable Cox proportional hazards model with time‐dependent covariates was fitted. The event of interest was treatment discontinuation; therefore, a hazard ratio lower than 1 indicates greater treatment persistence. Reaching the 14‐mg dose of oral semaglutide was associated with a clearly reduced risk of treatment discontinuation (HR 0.08, 95% CI 0.01–0.47). However, this effect decreased over time, as indicated by a significant interaction between dose escalation and follow‐up time (HR per month 1.16, 95% CI 1.03–1.31). Higher values of baseline HbA1c were independently associated with an increased risk of discontinuation, while obesity was associated with greater treatment persistence. No significant associations were observed for age, diabetes duration, or sex (Table 2).

**TABLE 2:** Multivariable Cox regression analysis for treatment persistence.

Covariate	HR	95% CI	*p* value
Sema 14 mg reached	**0.08**	0.01–0.47	**0.005**
Sema 14 mg × time	**1.16**	1.03–1.31	**0.012**
Age ≥ 65 yrs. (ref < 65 yrs.)	0.85	0.49–1.48	0.557
Baseline HbA1c (%)	**1.23**	1.02–1.48	**0.034**
Diabetes duration (yrs.)	0.98	0.95–1.02	0.392
BMI Overweight (ref NW)	0.72	0.36–1.44	0.355
BMI Obese (ref NW)	**0.47**	0.24–0.95	**0.036**
Sex Male (ref Female)	0.73	0.43–1.24	0.245

*Note:* HR refers to the risk of treatment discontinuation; HR < 1 indicates greater treatment persistence; ‘14 mg × time’ indicates the interaction between reaching 14 mg of semaglutide and follow‐up time, modelling the time‐dependent effect. Bold values indicate statistical significance (*p* < 0.05).

Abbreviations: BMI, body mass index; CI, confidence interval; HR, hazard ratio; NW, normal weight; ref., reference; Sema, semaglutide; yrs., years.

### 3.9 Safety and Withdrawal

No severe or documented hypoglycaemic episodes were reported. A total of 73 individuals (31.5%) discontinued oral semaglutide during the 24‐month study period. The reasons for discontinuation were gastrointestinal side effects (20.3%), limited effectiveness in lowering HbA1c and/or body weight (10.8%), patient preference (4.3%) and pregnancy (0.4%); unknown reasons accounted for the remaining 9 individuals (3.9%). Categories are not mutually exclusive, as more than one reason for discontinuation could be recorded for the same individual. Early discontinuation (≤ 3 months), mainly due to gastrointestinal adverse events, was observed in 11 individuals (4.7%).

### 3.10 Post‐Discontinuation Therapy and Outcomes After Switch

Of the 73 individuals who discontinued oral semaglutide, 58 (79.5%) were switched to another glucose‐lowering agent, while 15 (20.5%) did not initiate a replacement therapy. Within the group of switchers, the most common therapeutic strategy was the switch to another GLP‐1RA, which occurred in 21 individuals (36.2%), followed by SGLT‐2 inhibitors in 18 (31.0%), DPP‐4 inhibitors in 10 (17.2%) and basal insulin in 9 (15.5%). Among the 21 individuals switched to another GLP‐1RA, the most frequently prescribed molecules were injectable semaglutide once‐weekly (9, 42.9%), dulaglutide (9, 42.9%), and exenatide once‐weekly (3, 14.3%).

At baseline, individuals subsequently switched to another GLP‐1RA (GLP‐1RA switchers) and those who continued oral semaglutide were comparable in terms of HbA1c (7.88% ± 1.33% vs. 8.02% ± 1.38%; *p* = 0.688) and body weight (83.2 ± 17.6 vs. 79.7 ± 21.1 kg; *p* = 0.530). In linear mixed‐effects models with a time × switcher‐status interaction, changes in HbA1c over time did not differ significantly between subsequent GLP‐1RA switchers and non‐switchers (interaction *p* = 0.208 to 0.856 across timepoints). Body weight, in contrast, declined significantly less over time in switchers compared with non‐switchers (interaction *β* = +2.05 kg per time unit; *p* = 0.005).

In GLP‐1RA switchers, HbA1c decreased significantly from 8.21% ± 1.44% immediately before the switch to 7.23% ± 0.87% after the switch (median time between measurements 6 months; paired *t*‐test, Δ = −0.98 percentage points, 95% CI −1.63 to −0.33; *p* = 0.005); in the same group, a mean reduction of 3.06 ± 5.64 kg of body weight was observed, which did not reach statistical significance (95% CI −0.72 to +6.85; *p* = 0.10).

## 4 Discussion

In this real‐world, single‐centre retrospective study, oral semaglutide was associated with sustained reductions in HbA1c and body weight, a progressive increase in the composite endpoint of glycaemic and weight control, stable renal function, an improved lipid profile, and treatment persistence of 68.5% over a 24‐month follow‐up. This analysis extends our previous 6‐month observation [3] to 2 years, adding to the still limited real‐world evidence on oral semaglutide beyond 18 months of follow‐up [4].

The magnitude of the glycaemic and weight effects observed at 24 months in our cohort (HbA1c −0.8%; body weight −5.7 kg) is consistent with the range reported in the PIONEER randomised programme [1] and in shorter real‐world cohorts, in which HbA1c reductions ranging from −0.4% to −1.8% and body weight reductions from −2 to −9 kg have been described [4, 5, 6, 7, 8]. Importantly, both effects were evident at the first follow‐up and were maintained throughout the 2‐year observation period (Figure 1). In parallel, the proportion of individuals achieving the composite endpoint of HbA1c ≤ 7% and weight loss ≥ 5% nearly doubled from 23.6% at 6 months to 44.0% at 24 months. The accumulation of clinically relevant benefit should however be interpreted with caution, since longitudinal analysis was restricted to individuals remaining on treatment and may therefore reflect, at least in part, a survivor effect related to selective persistence of responders.

An unexpected finding emerged from the sub‐analysis on the timing of oral semaglutide administration. Contrary to the initial concern that non‐breakfast administration might compromise fasting conditions and reduce effectiveness, individuals taking the drug before lunch or dinner had significantly lower HbA1c levels over the 24‐month follow‐up, an effect that persisted after adjustment for baseline BMI, age, and sex. This observation may suggest that acknowledging patient preferences through attentive listening and open patient–physician dialogue translates into improved adherence.

The stability of renal function observed over the 2‐year follow‐up deserves specific consideration. The estimated mean change in eGFR of −0.28 mL/min/1.73 m^2^ at 24 months is substantially smaller than the annual decline of 1–3 mL/min/1.73 m^2^ that would be expected in a population with the characteristics of our cohort (i.e., median age 67 years, median diabetes duration 9 years, and approximately one in five individuals with CKD stage III at baseline). Two complementary explanations may account for this observation. First, this pattern may reflect a direct nephroprotective effect of GLP‐1RAs, recently supported for oral semaglutide by the SOUL trial [2] and for subcutaneous semaglutide in the FLOW trial [9]. Second, the proportion of individuals receiving an SGLT‐2i inhibitor increased from approximately 40% at baseline to 50% at 24 months; given the well‐established nephroprotective effect of this drug class, an additive contribution to the observed renal stability cannot be excluded. UACR showed no significant variation over the follow‐up, a finding directionally consistent with the eGFR data, although the wide confidence intervals and the skewed distribution of this parameter warrant cautious interpretation. In addition, total and LDL cholesterol also decreased significantly, whereas HDL cholesterol and triglycerides remained stable, with background lipid‐lowering therapies unchanged throughout follow‐up (data not shown), consistently with the metabolic profile of the GLP‐1RA class [10, 11, 12].

A distinctive contribution of the present analysis is the evaluation of treatment persistence over 2 years, an endpoint of major clinical and pharmacoeconomic relevance for chronic therapies. At 24 months, 68.5% of individuals were still on treatment with oral semaglutide, with a mean estimated persistence time of 19.4 months, a figure that compares favourably with persistence rates reported for oral GLP‐1RAs and other oral antihyperglycaemic agents in real‐world cohorts [4, 13].

Approximately half of the individuals still on therapy at the end of follow‐up were receiving the highest dose of 14 mg/day; nonetheless, the longitudinal trajectories of HbA1c and body weight did not differ significantly between those reaching the 14 mg dose and those remaining on lower doses. This pattern is consistent with the dose–response data from the PIONEER programme [1], in which incremental benefits at the 14 mg dose are real but modest, and supports an individualised, tolerability‐driven titration strategy rather than systematic up‐titration to the maximum dose.

In the multivariable analysis of factors associated with treatment persistence, escalation to the 14‐mg dose was associated with a lower risk of discontinuation, although this association weakened over time. This finding likely reflects a selection of individuals who tolerated treatment and perceived a sustained benefit, rather than a direct effect of higher dosing per se. Higher baseline HbA1c was associated with earlier discontinuation, whereas baseline obesity was associated with greater persistence. These associations may reflect differences in perceived treatment benefit: weight reduction is perceived by patients as a tangible treatment goal that may reinforce adherence to therapy, whereas HbA1c may be perceived as a less compelling therapeutic goal from the patient's perspective, potentially contributing to earlier discontinuation.

According to this consideration, overall, 31.5% of the cohort discontinued oral semaglutide within 24 months, mainly for adverse events rather than for lack of efficacy. Among discontinuers with follow‐up data, the switch to another GLP‐1RA, most frequently injectable semaglutide once‐weekly or dulaglutide, was the most common strategy. Switchers and non‐switchers were comparable at baseline for HbA1c and body weight, as well as for HbA1c trajectories during oral semaglutide treatment. Body weight, however, decreased significantly less over time in switchers, suggesting that a less pronounced weight response, combined with gastrointestinal intolerance, may have contributed to the decision to switch. After the switch, HbA1c further decreased significantly, with a non‐significant trend towards additional weight loss. These findings suggest that the class effect of GLP‐1 receptor agonism can be maintained beyond the initial oral formulation, and that intolerance to oral semaglutide should not preclude continued use of the class.

The safety profile observed over 24 months was reassuring and fully consistent with the known characteristics of the GLP‐1RA class: no severe or documented hypoglycaemic episodes were recorded, and the rate of discontinuation due to gastrointestinal intolerance (20.3%) is in line with that reported in both the PIONEER trials and previous real‐world cohorts [1, 7]. In keeping with these data, some discontinuations occurred within the first 3 months of treatment, reinforcing the well‐established notion that careful patient selection, structured counselling on the unique administration requirements of oral semaglutide, and gradual dose escalation are pivotal to optimise tolerability and long‐term retention in routine clinical care.

A bioequivalent second‐generation formulation of oral semaglutide, recently shown to be bioequivalent to the first‐generation product at lower doses [14], is progressively replacing the first‐generation product in clinical practice, and the real‐world data presented here can reasonably be expected to translate to the 2G formulation.

Several limitations should be acknowledged. First, the retrospective, single‐centre design and the absence of a control group preclude causal inference, and residual confounding by indication and by changes in concomitant therapies cannot be ruled out. Second, longitudinal estimates of effectiveness, including the composite endpoint of HbA1c and weight control at each timepoint, are based on individuals remaining on treatment and may therefore overestimate the average effect at the whole‐cohort level, since patients who had discontinued therapy are not represented in the denominator. Third, the sub‐analysis on administration timing was conducted on a small non‐breakfast subgroup and may be affected by self‐selection of particularly adherent patients, and its interpretation should therefore be considered hypothesis‐generating. Fourth, structured information on lifestyle modifications, adherence to drug administration instructions, and patient‐reported outcomes was not systematically captured. Finally, generalisability to other healthcare settings and to populations with different ethnic and clinical case‐mix should be tested in independent cohorts. Notwithstanding these limitations, the use of standardised electronic medical records, the inclusion of a clinically representative cohort, and a follow‐up extending well beyond that of most published real‐world series strengthen the external validity of our observations.

In conclusion, oral semaglutide provided sustained glycaemic, weight, and lipid benefits, a progressive increase in composite treatment success, stable renal function, and a treatment persistence of 68.5% over 2 years in routine specialist care.

## Author Contributions

Conceptualisation: Gloria Formoso and Maria Pompea Antonia Baldassarre. Methodology and formal analysis: Giulia Di Dalmazi. Investigation: Maria Pompea Antonia Baldassarre, Federica Carrieri, Giorgia Centorame, and Luigi Piacentino. Writing – original draft preparation: Maria Pompea Antonia Baldassarreand, Giulia Di Dalmazi, and Federica Carrieri. Writing – review and editing: Gloria Formoso and Maria Pompea Antonia Baldassarre. All authors have read and agreed to the published version of the manuscript.

## Funding

The authors have nothing to report.

## Conflicts of Interest

The authors declare no conflicts of interest.

## Data Availability

The data that support the findings of this study are available from the corresponding author upon reasonable request. The data are not publicly available due to privacy and ethical restrictions related to clinical patient information.
